# Reliability of a rapid hematology stain for sputum cytology*

**DOI:** 10.1590/S1806-37132014000300008

**Published:** 2014

**Authors:** Jéssica Gonçalves, Emilio Pizzichini, Marcia Margaret Menezes Pizzichini, Leila John Marques Steidle, Cristiane Cinara Rocha, Samira Cardoso Ferreira, Célia Tânia Zimmermann

**Affiliations:** Clinical Analysis Department, Federal University of Santa Catarina University Hospital, Florianópolis, Brazil; Federal University of Santa Catarina University Hospital, Florianópolis, Brazil; Graduate Program in Medical Sciences, Federal University of Santa Catarina University Hospital, Florianópolis, Brazil; Department of Clinical Medicine, Federal University of Santa Catarina University Hospital, Florianópolis, Brazil; Center for Research on Asthma and Airway Inflammation, Federal University of Santa Catarina University Hospital, Florianópolis, Brazil; Clinical Analysis Department, Federal University of Santa Catarina University Hospital, Florianópolis, Brazil; Clinical Analysis Department, Federal University of Santa Catarina University Hospital, Florianópolis, Brazil

**Keywords:** Sputum, Sputum, Azure stains

## Abstract

**Objective:**

To determine the reliability of a rapid hematology stain for the cytological analysis of induced sputum samples.

**Methods:**

This was a cross-sectional study comparing the standard technique (May-Grünwald-Giemsa stain) with a rapid hematology stain (Diff-Quik). Of the 50 subjects included in the study, 21 had asthma, 19 had COPD, and 10 were healthy (controls). From the induced sputum samples collected, we prepared four slides: two were stained with May-Grünwald-Giemsa, and two were stained with Diff-Quik. The slides were read independently by two trained researchers blinded to the identification of the slides. The reliability for cell counting using the two techniques was evaluated by determining the intraclass correlation coefficients (ICCs) for intraobserver and interobserver agreement. Agreement in the identification of neutrophilic and eosinophilic sputum between the observers and between the stains was evaluated with kappa statistics.

**Results:**

In our comparison of the two staining techniques, the ICCs indicated almost perfect interobserver agreement for neutrophil, eosinophil, and macrophage counts (ICC: 0.98-1.00), as well as substantial agreement for lymphocyte counts (ICC: 0.76-0.83). Intraobserver agreement was almost perfect for neutrophil, eosinophil, and macrophage counts (ICC: 0.96-0.99), whereas it was moderate to substantial for lymphocyte counts (ICC = 0.65 and 0.75 for the two observers, respectively). Interobserver agreement for the identification of eosinophilic and neutrophilic sputum using the two techniques ranged from substantial to almost perfect (kappa range: 0.91-1.00).

**Conclusions:**

The use of Diff-Quik can be considered a reliable alternative for the processing of sputum samples.

## Introduction

The understanding of the mechanisms of diseases and their correct diagnosis has been made possible by analysis of body fluids in several areas of medicine. In the past, sputum analysis was considered not to be reliable or reproducible enough to assist in the understanding of the mechanisms of respiratory diseases.^(^ ^1^ ^)^ More recently, significant advances related to the processing of sputum samples have allowed the method of sputum examination to become feasible, reproducible, valid, and responsive to interventions. Several researchers have used this method to study the various aspects of airway inflammation in asthma. The use of sputum examination has been further extended to COPD, cystic fibrosis, chronic cough, idiopathic pulmonary fibrosis, and other respiratory diseases.

Inflammation is central in the pathogenesis of airway diseases and is considered responsible for their symptoms, airflow obstruction, exacerbations, and secondary structural changes.^(^ ^2^ ^)^ Therefore, airway inflammation plays an extremely significant role in two major obstructive respiratory tract diseases: asthma and COPD.^(^ ^3^ ^)^ Both in asthma and in COPD, there is great heterogeneity in clinical and inflammatory characteristics, which result in different clinical phenotypes.^(^ ^4^ ^)^ Consequently, there is a need to characterize the phenotype of patients in order to optimize their clinical management, particularly in more severe cases.^(^ ^4^ ^-^ ^6^ ^)^

Examination of induced sputum is currently considered reliable, reproducible, discriminative of different types of inflammation, and responsive to interventions. Therefore, it has been an important tool in the management of inflammatory diseases of the airways.^(^ ^7^ ^)^ In addition, sputum induction is a safe minimally invasive technique,^(^ ^8^ ^)^ making it possible to identify the type of inflammation and its intensity.^(^ ^9^ ^,^ ^10^ ^)^ However, its use in clinical practice is still very restricted because of the method for sputum induction and for the processing of sputum samples, which is laborious and time-consuming and requires highly trained personnel.

Induced sputum slides for differential cell counts, both in research and in clinical practice, have been stained with May-Grünwald-Giemsa or with Wright-Giemsa. These stains are part of a group called "Romanowsky stains".^(^ ^11^ ^)^ Diff-Quik (a rapid hematology stain), which is also based on the Romanowsky technique,^(^ ^12^ ^)^ uses similar reagents, but the staining time is considerably shorter; whereas the standard stain requires 34 min, the rapid stain is performed within 2 min.

Shortening the total processing time would be extremely important for improving the viability of the technique. In addition, the cost of Diff-Quik is considerably lower than that of the standard stain. Therefore, the validation of this technique is important, especially for developing countries, such as Brazil.

The objective of the present study was to assess the reliability of Diff-Quik for the cytological analysis of induced sputum samples.

## Methods

The study included 50 patients, of whom 21 were adult patients with uncontrolled asthma, characterized by an Asthma Control Questionnaire^(^ ^13^ ^)^ score greater than 1.7 in the previous week and objectively confirmed (in the 3 previous years) by reversible airflow limitation (a > 12% increase in FEV_1_ and a > 200 mL increase in FEV_1_ after inhalation of a short-acting bronchodilator) in participants with airflow limitation (an FEV_1_/FVC ratio < 0.7); 19 COPD patients aged > 40 years who had a history of respiratory symptoms associated with moderate or severe airflow obstruction (an FEV_1_ < 50% of predicted and an FEV_1_/FVC ratio < 0.7), were receiving any type of medication for COPD, and were (current or former) heavy smokers with a smoking history of > 20 pack-years; and 10 healthy nonsmokers who had no respiratory symptoms and whose diagnostic status was objectively confirmed by normal spirometry results. The study excluded patients who had respiratory infection in the four previous weeks, those who had severe diseases of other systems, those who had other known pulmonary diseases, and pregnant women.

The study was conducted at the Center for Research on Asthma and Airway Inflammation, located at the Federal University of Santa Catarina University Hospital in the city of Florianópolis, Brazil, and was approved by the local Human Research Ethics Committee (Process no. 2093; FR 437236, issued on November 28, 2011). All participants gave written informed consent after being given a detailed explanation of the study.

Participants underwent pre- and post-bronchodilator spirometry with a computerized spirometer (Koko; PDS Instrumentation, Inc., Louisville, CO, USA), in accordance with the American Thoracic Society guidelines^(^ ^14^ ^)^ The reference values used were those of Crapo et al.^(^ ^15^ ^)^

Subsequently, sputum induction was performed in accordance with the method described by Pizzichini et al.^(^ ^16^ ^)^ The procedure involved inhalation of an isotonic saline aerosol (0.9%) followed by serial inhalation of increasing concentrations of hypertonic saline aerosol (3%, 4%, and 5%) via a Fisoneb ultrasonic nebulizer (Fisons, Pickering, Ontario, Canada). Aerosol inhalation was continued for 1-2 min, according to the level of bronchoconstriction severity before the procedure, and was followed by measurement of FEV_1_. Participants were instructed to rinse their mouths with water, swallow the water, and blow their noses in order to reduce contamination by saliva or postnasal discharge. They were then asked to cough and expectorate the sputum into a clean container. These procedures were repeated consecutively, with the solution concentration being increased every 7 min for 21 min or until there was a ≥ 20% decrease in FEV_1_.

The processing of sputum samples was started within 2 h of collection, which is the longest time reported in the literature.^(^ ^2^ ^)^ The thick portions of the expectorated material were selected with the naked eye or under visualization with an inverted microscope, and the sputum was separated from the saliva. The selected fractions were treated with 0.1% DTT at a ratio of four times the fraction volume. This mixture was homogenized with a Pasteur pipette and agitated on a desktop shaker for 15 min. Dulbecco's PBS was added thereto in an amount that was four times the initial volume of sputum selected, and the resulting suspension was filtered to remove cell debris and undissolved mucus. Subsequently, total leukocyte counts were performed with a modified Neubauer hemocytometer, excluding squamous cells. Cell viability was determined by the trypan blue exclusion test. The sample was adjusted to 1.0 × 106 cells/mL, and we prepared four slides, which were coded. In the present study, all of the slides were prepared using the cytocentrifugation method (cytospin). After air-drying, two slides were stained with May-Grünwald-Giemsa, and the other two were stained with Diff-Quik (the technique under study).

May-Grünwald-Giemsa staining was performed with an automated system (Sysmex sp1000iTM; Sysmex Co., Kobe, Japan). For this technique, the slides were fixed by immersion in analytical grade methanol, and then they were immersed in a May-Grünwald stain solution and a dilute May-Grünwald solution (1:1). Immediately afterward, the slides were immersed in a freshly prepared Giemsa stain solution (dilution 1:10) and subsequently dried. The whole procedure lasted exactly 34 min, as recommended by the equipment manufacturer.

Diff-Quik was performed manually. The process included initial immersion of the slides in solution no. 1 (0.1% triarylmethane), moving up and down continuously for 5-10 seconds (5-10 one-second immersions). Subsequently, extensions were immersed in solution no. 2 (0.1% xanthene), repeating the same procedure. After draining, the slides were immersed in solution no. 3 (0.1% thiazine), repeating the same procedure. The slides were rinsed with distilled water and allowed to air-dry.^(^ ^12^ ^)^ This staining procedure took a maximum of 2 min to complete. Two researchers, trained in reading induced sputum slides, independently counted 400 non-squamous cells on the slides stained either with May-Grünwald-Giemsa or Diff-Quik. Because the slides were coded, the slide readers were prevented from identifying their respective pairs or previous reading results.

The reliability for cell counting using the two techniques (standard stain vs. tested stain) was evaluated by determining the intraclass correlation coefficients (ICCs) for intraobserver and interobserver agreement, and Bland & Altman plots were used.^(^ ^17^ ^)^ The interpretation of ICCs was based on the classification proposed by Landis & Koch.^(^ ^18^ ^)^ Agreement in the identification of eosinophilic sputum (eosinophils ≥ 3%)^(^ ^6^ ^)^ and neutrophilic sputum (neutrophils > 64%)^(^ ^19^ ^)^ between the observers and between the stains was evaluated with kappa statistics.^(^ ^18^ ^)^ Differences between the characteristics of the groups studied were examined by ANOVA and the Bonferroni test in the post hoc analysis. The statistical tests were two-tailed, and the level of significance was set at 5%. The Statistical Package for the Social Sciences, version 18.0 (SPSS Inc., Chicago, IL, USA) was used for the analyses.

## Results

Sputum induction was performed in 62 individuals, 50 (80.6%) of whom were able to produce an adequate sample, with cell viability greater than 50%. Twelve induced sputum samples were considered inadequate because of excessive salivary contamination (> 20% of squamous cells), low cell viability (< 50%), or insufficient material to prepare the slides. The demographic, clinical, and functional characteristics of the participants are shown in Table 1. The groups were distinct and well characterized, as demonstrated by their demographic, clinical, and functional characteristics.

**Table 1:** - Demographic, clinical, and functional characteristics of the participants.a

Characteristic	Groups			p
	Asthma	COPD	Control	
	(n = 21)	(n = 19)	(n = 10)	
Age, years^b^	47.3 (22-68)	62.8 (52-77)	38.4 (21-58)	< 0.001*.*** and 0.2**
Female gender^c^	12 (57.0)	5 (26.3)	7 (70.0)	0.03
Pre-BD FEV1,% of predicted	55.3 ± 11.9	50.2 ± 18.2	102.2 ± 7.8	0.1* and < 0.001**.***
Post-BD FEV1,% of predicted	64.9 ± 11.7	52.7 ± 18.2	104.5 ± 8.7	0.02* and < 0.001**.***
Pre-BD FEV1/FVC, %	58.3 ± 9.5	52.5± 13.8	80.4 ± 5.0	0.4* and < 0.001**.***
Post-BD FEV1/FVC,%	61.3 ± 9.2	53.2 ± 14.4	82.4 ± 4.6	0.06* and < 0.001**.***
Pre-BD ΔFEV1, L	0.29 ± 0.29	0.07 ± 0.09	0.08 ± 0.08	0.003*, 0.02**, and 1.0***
Post-BD ΔFEV1, %	19.1 ± 17.6	6.1 ± 7.4	2.5 ± 2.4	0.005*, 0.002**, and 1.0***

Pré-BD : antes do uso de broncodilatador

pós-BD : depois do uso de broncodilatador

a Valores expressos em média ± dp, exceto onde indicado.

b Valor expresso em média (mínimo-máximo).

c Valor expresso em n (%)

* Grupo asma vs. grupo DPOC

** Grupo asma vs. controle

*** Grupo DPOC vs. controle

**Figure 1:**
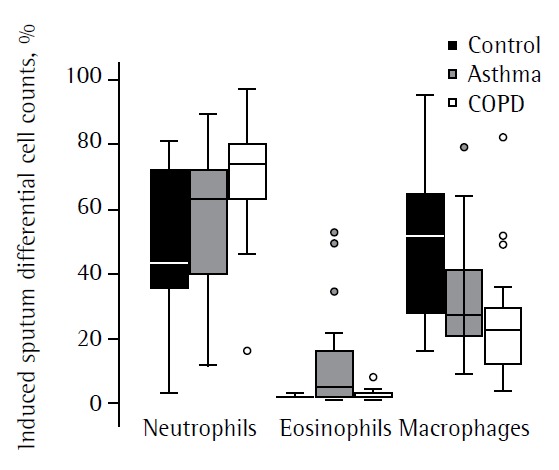
Induced sputum differential cell counts in the three groups studied.

**Figure 2:**
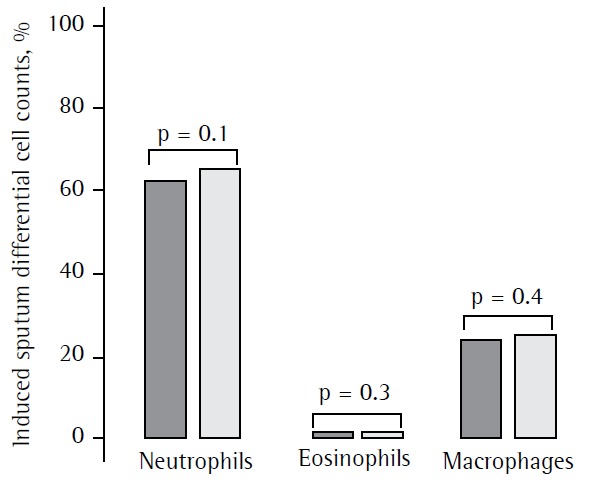
Median differential cell counts on the cytospin slides stained either with May-Grünwald-Giemsa (dark gray bar) or Diff-Quik (light gray bar)

The cellular characteristics of induced sputum were as expected for the different groups studied. The sputum of asthma patients was characterized by a significantly higher proportion of eosinophils than that found in the sputum of COPD patients and healthy controls. In contrast, the sputum of COPD patients showed a significant increase in total cell counts and in the proportion of neutrophils when compared with that of controls. The control group showed a significantly higher proportion of macrophages than did the other two groups. Figure 1 shows the proportions of neutrophils, eosinophils, and macrophages in the different groups studied. In Figure 2, the median proportions of neutrophils, eosinophils, and macrophages, in the study sample as a whole, are separated by type of stain used. No significant differences were found for the cell counts on the slides stained by either of the two techniques used.

The results for interobserver agreement for induced sputum differential cell counts on the cytospin slides stained by the May-Grünwald-Giemsa technique show that the medians and percentiles were similar between the two observers, and the ICCs indicated almost perfect agreement for eosinophil, neutrophil, and macrophage counts (ICC = 1.00, 0.99, and 0.98, respectively). Interobserver agreement for lymphocyte counts was substantial (ICC = 0.76). For the slides stained by the Diff-Quik technique, the medians and percentiles were also very close between the two observers, and the ICC indicated almost perfect interobserver agreement for eosinophil, neutrophil, macrophage, and lymphocyte counts (ICC = 1.00, 0.99, 0.99, and 0.83, respectively).

**Figure 3:**
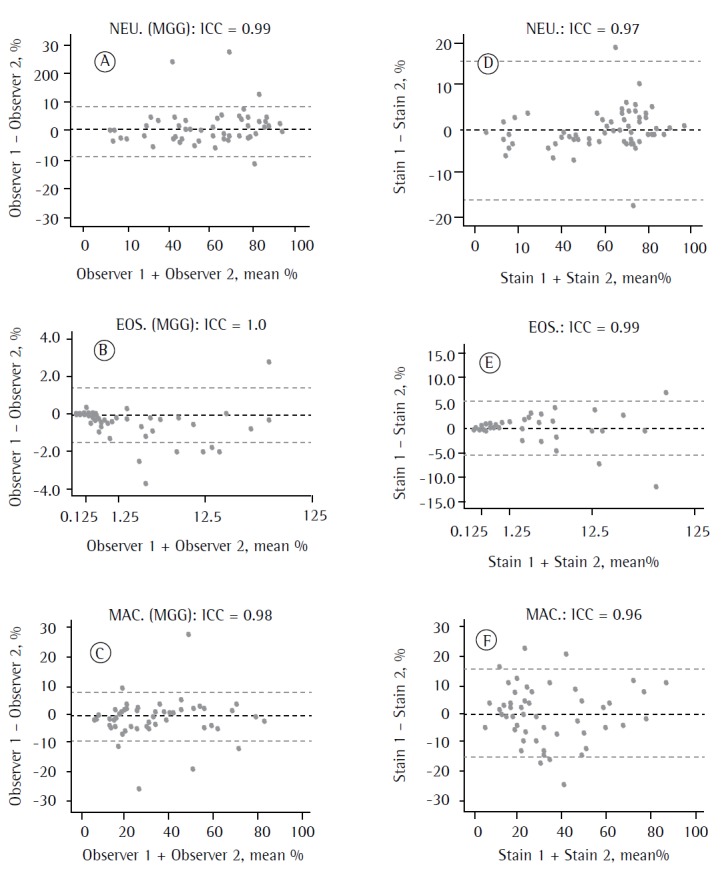
Bland & Altman plots. Interobserver reproducibility for the proportion of neutrophils (in A), eosinophils (in B), and macrophages (in C) on the cytospin slides prepared from induced sputum samples and stained with May-Grünwald-Giemsa (MGG). Intraobserver reproducibility for the proportion of neutrophils (in D), eosinophils (in E), and macrophages (in F) on the cytospin slides prepared from induced sputum samples and stained by either of the two staining techniques. The plots refer to the differences between the readings by observers 1 and 2 (y axis) in relation to the mean readings by observers 1 and 2 (x axis). The central broken line indicates absence of differences, and the peripheral broken lines indicate two standard deviations of the mean of the differences. ICC: intraclass correlation coefficient; NEU.: neutrophils; EOS.: eosinophils; and MAC.: macrophages.

Regarding intraobserver agreement, the ICC values for the two observers for the differential cell counts on the pairs of cytospin slides stained by either of the two studied techniques indicated that it was almost perfect for neutrophils (ICC = 0.97 for both), eosinophils (ICC = 0.99 and 0.98), and macrophages (ICC = 0.96 for both). For lymphocyte counts, intraobserver agreement was substantial for observer 1 (ICC = 0.75) and moderate for observer 2 (ICC = 0.65). These results are shown graphically in Figure 3.^(^ ^17^ ^)^

Interobserver agreement for the identification of eosinophilic and neutrophilic sputum using the two techniques ranged from substantial to almost perfect, as shown in Table 2. However, although intraobserver agreement was substantial, it was lower than was interobserver agreement.

**Table 2:** - Interobserver and intraobserver agreement for the identification of eosinophilic and neutrophilic sputum in the evaluation of slides stained by either of the two studied techniques.

Interobserver agreement	Sputum type	kappa	p
Diff-Quik stain	Eosinophilic	1.000	< 0.001
	Neutrophilic	1.000	< 0.001
May-Grünwald-Giemsa stain	Eosinophilic	0.905	< 0.001
	Neutrophilic	0.960	< 0.001
Intraobserver agreement	Sputum type	kappa	p
Observer 1	Eosinophilic	0.746	< 0.001
	Neutrophilic	0.801	< 0.001
Observer 2	Eosinophilic	0.758	< 0.001
	Neutrophilic	0.760	< 0.001

## Discussion

The results of the present study show that cytospin slides stained either by the May-Grünwald-Giemsa technique or with Diff-Quik yield similar cell counts, with high intraobserver and intraobserver agreement. These results demonstrate the reliability of the Diff-Quik technique for use in the processing of induced sputum samples. This fact is relevant because the Diff-Quik technique is simpler, allows a reduction in sample processing time of up to 32 min without impairing sample quality, and is considerably cheaper.

To our knowledge, this was the first study to evaluate the reliability of the Diff-Quik technique for use in induced sputum cytology, by comparing it with a standard staining technique, i.e., the May-Grünwald-Giemsa technique. It is important to evaluate the reliability and reproducibility of the results in order to confirm the accuracy of the results obtained by using Diff-Quik. In the present study, the reliability of Diff-Quik was tested by two distinct strategies. The first strategy was to calculate the ICCs for the cell counts performed by two independent observers, blinded to the identification of the slides. Although the stains used in the present study could be identified by the appearance of the slides, differing codes were used to prevent the identification of the respective pairs of slides. Previous studies^(^ ^20^ ^-^ ^22^ ^)^ have shown that intraobserver and interobserver agreement for cell counts on cytospin slides stained with Wright-Giemsa and May-Grünwald-Giemsa could be considered perfect, but that it depended on the degree of salivary contamination on the slides.^(^ ^21^ ^)^ The results obtained with Diff-Quik in the present study are in line with those of the aforementioned studies. The second strategy was to examine the reliability of Diff-Quik for identifying eosinophilic sputum and neutrophilic sputum. This is relevant because, in clinical practice, sputum examination is used to identify phenotypes of severe asthma, predict response to treatment, and decrease the number of asthma exacerbations by control of eosinophilic inflammation.

Intraobserver agreement for the identification of eosinophilic and neutrophilic sputum by the two staining techniques was found to be substantial. In addition, interobserver agreement for the identification of eosinophilic and neutrophilic sputum was almost perfect. These results again demonstrate the reliability of Diff-Quik, because they confirm its accuracy for identifying the different inflammatory phenotypes. However, the results also showed that interobserver agreement was higher than intraobserver agreement for the identification of the phenotypes. This difference could be due to variability in cell content on the slides stained by either of the two techniques. Although intraobserver agreement was substantial, this particular result suggests caution and a need for further studies to identify the reason for this variability.

Interobserver reproducibility for differential leukocyte counts in induced sputum samples has been previously reported.^(^ ^20^ ^-^ ^22^ ^)^ In 1997, one group of authors reported high interobserver reproducibility for all cell types studied. Those authors also found lower agreement for lymphocytes than for the other cell types. The lower agreement for the proportions of lymphocytes was considered to be due to the very small amount of this cell type in the induced sputum samples. In the present study, we also found lower agreement for lymphocyte counts. However, this was not emphasized because it is a known fact that these variations are not clinically relevant.

In one study,^(^ ^21^ ^)^ there was good interobserver agreement for neutrophil, eosinophil, and macrophage counts, and, again, this agreement was lower for lymphocyte counts; the justification for the lower repeatability rate was related not only to the scarcity of lymphocytes in the samples but also to the difficulty in identifying these cells. One group of authors^(^ ^21^ ^)^ confirmed the results of a previous study,^(^ ^20^ ^)^ demonstrating that the reproducibility of induced sputum differential cell counts is affected by salivary contamination and low cell viability. In the present study, samples with > 20% salivary contamination or < 50% viability were considered inadequate for analysis.

Methods for refinement of sputum examination have greatly contributed to its accuracy and reproducibility.^(^ ^23^ ^)^ However, previous studies have primarily focused on the steps preceding the preparation of cytospin slides and the liquid phase of sputum.^(^ ^24^ ^-^ ^27^ ^)^

In 2003, a study comparing the results and costs of three techniques for analysis of induced sputum samples was published.^(^ ^28^ ^)^ The techniques consisted of one of the following: preparation of smears from sputum not treated with DTT; preparation of smears from sputum treated with DTT; or preparation of cytospin slides from sputum treated with DTT. Although the first two techniques reduced the time and cost of sputum sample analysis, Spearman's correlation coefficient, which ranged from 0.57 to 0.64 for eosinophil counts and from 0.51 to 0.57 for neutrophil counts ((p < 0.01 for both), can be considered inadequate for these types of techniques. The authors concluded that the technique that uses cytospin slides prepared from samples treated with DTT is more suitable for research purposes and for use in specialized centers.^(^ ^28^ ^)^

Some of the aspects that hinder the widespread use of induced sputum are its complexity and the sample processing time. Two previous studies have attempted to reduce the complexity of sample processing,^(^ ^29^ ^,^ ^30^ ^)^ both using a sputum filtration device (Accufilter; Cellometrics, Hamilton, Ontario, Canada), which would be an alternative for standardizing the processing of induced sputum samples. The device consists of a kit with a tube used for weighing and treating sputum selected from saliva, connected to a filter and to a reception tube containing DTT, saline solution, and trypan blue. One of those studies^(^ ^30^ ^)^ sought to determine the validity of using this device in the analysis of sputum samples, by comparing it with the standard method. The study reported ICCs that indicate good reproducibility for eosinophil and neutrophil counts between the methods; however, the ICCs indicated a reduction in cell viability and total cell counts, as well as an increase in the proportion of squamous epithelial cells, with the use of that device.

In summary, the high degree of agreement for the cell counts on the cytospin slides stained either by the May-Grünwald-Giemsa technique or with Diff-Quik attests to the reliability of the latter stain, which justifies the recommendation that it be used when the objective is to reduce sample processing time and induced sputum costs.
