# Supplementary material for: Reliability of a rapid hematology stain for sputum cytology
Source: J Bras Pneumol. 2014 May-Jun;40(3):250–8. doi: 10.1590/S1806-37132014000300008 (PMC4109197; doi:10.1590/S1806-37132014000300008)
Supplement: Translation

# Translation: Confiabilidade da coloração hematológica rápida para citologia de escarro*

**Authors:** Jéssica Gonçalves, Emilio Pizzichini, Marcia Margaret Menezes Pizzichini, Leila John Marques Steidle, Cristiane Cinara Rocha, Samira Cardoso Ferreira, Célia Tânia Zimmermann

**Affiliations:** Serviço de Análises Clínicas, Hospital Universitário, Universidade Federal de Santa Catarina, Florianópolis (SC) Brasil; Universidade Federal de Santa Catarina, Florianópolis (SC) Brasil; Programa de Pós-Graduação em Ciências Médicas, Universidade Federal de Santa Catarina, Florianópolis (SC) Brasil; Departamento de Clínica Médica, Universidade Federal de Santa Catarina, Florianópolis (SC) Brasil; Núcleo de Pesquisa em Asma e Inflamação das Vias Aéreas, Hospital Universitário, Universidade Federal de Santa Catarina, Florianópolis (SC) Brasil; Serviço de Análises Clínicas, Hospital Universitário, Universidade Federal de Santa Catarina, Florianópolis (SC) Brasil; Serviço de Análises Clínicas, Hospital Universitário, Universidade Federal de Santa Catarina, Florianópolis (SC) Brasil

**Keywords:** Escarro\análise, Escarro\citologia, Corantes azur

## Abstract

**Objetivo:**

Determinar a confiabilidade da coloração hematológica rápida para a análise do escarro induzido.

**Métodos:**

Estudo transversal comparando a técnica padrão (coloração May-Grünwald-Giemsa) com a coloração hematológica rápida (panótico rápido). Participaram do estudo 50 indivíduos (21 asmáticos, 19 portadores de DPOC e 10 controles). Após a coleta do escarro induzido, foram preparadas 4 lâminas, sendo 2 coradas por May-Grünwald-Giemsa e 2 por panótico rápido. As lâminas foram lidas de forma independente por dois pesquisadores capacitados para o exame de escarro induzido e cegados para a identificação das lâminas. A confiabilidade para as contagens celulares dos dois métodos foi avaliada pela determinação dos coeficientes de correlação intraclasse (CCI) para as concordâncias intraobservador e interobservador. As concordâncias na identificação de escarro neutrofílico e eosinofílico entre observadores e entre as duas colorações foram calculadas por estatística kappa.

**Resultados:**

Nas duas colorações, os CCI apontaram concordância interobservador quase perfeita para as contagens de neutrófilos, eosinófilos e macrófagos (variação do CCI: 0,98-1,00) e substancial para as contagens de linfócitos (variação do CCI: 0,76-0,83). Na análise intraobservador, a concordância foi quase perfeita para as contagens de neutrófilos, eosinófilos e macrófagos (variação do CCI: 0,96-0,99) e de moderada a substancial para as contagens de linfócitos (CCI = 0,65 e 0,75 para observadores 1 e 2, respectivamente). A concordância interobservador na identificação de escarro eosinofílico e neutrofílico para os dois métodos de coloração variou entre substancial e quase perfeita (variação kappa: 0,91-1,00).

**Conclusões:**

O panótico rápido pode ser considerado uma alternativa confiável para o processamento de amostras de escarro.

## Introdução

A compreensão dos mecanismos das doenças e o seu diagnóstico correto tem sido possibilitada pela análise de fluídos corporais em diversas áreas da medicina. No passado, a análise do escarro era considerada pouco confiável e pouco reprodutível para auxiliar no entendimento dos mecanismos das doenças respiratórias.^(^ ^1^ ^)^ Mais recentemente, avanços importantes ligados ao processamento do escarro permitiram que a metodologia desse exame se tornasse exequível, reprodutível, válida e responsiva às intervenções. Vários pesquisadores têm utilizado esse método para estudar os diversos aspectos da inflamação das vias aéreas na asma. A utilização do exame do escarro foi estendida ainda para a DPOC, fibrose cística, tosse crônica, fibrose pulmonar idiopática e outras doenças respiratórias.

A inflamação é central na patogênese das doenças das vias aéreas e é considerada responsável por seus sintomas, obstrução ao fluxo de ar, exacerbações e alterações estruturais secundárias.^(^ ^2^ ^)^ Por isso, a inflamação das vias aéreas tem um papel extremamente significativo nas duas principais doenças obstrutivas do trato respiratório: asma e DPOC.^(^ ^3^ ^)^ Tanto na asma quanto na DPOC há uma grande heterogeneidade nas características clínicas e inflamatórias, que resultam em diferentes fenótipos clínicos.^(^ ^4^ ^)^ Por esse motivo, existe a necessidade de se caracterizar o fenótipo do paciente para otimizar seu manejo clínico, particularmente nos casos mais graves.^(^ ^4^ ^-^ ^6^ ^)^

Atualmente, o exame do escarro induzido é considerado confiável, reprodutível, discriminativo de diferentes tipos de inflamação e responsivo às intervenções. Por isso, tem sido um instrumento importante no manejo das doenças inflamatórias das vias aéreas.^(^ ^7^ ^)^ Além disso, a indução do escarro é uma técnica segura e pouco invasiva,^(^ ^8^ ^)^ permitindo identificar o tipo de inflamação e sua intensidade. ^(^ ^9^ ^,^ ^10^ ^)^ Contudo, seu uso na prática clínica ainda é muito restrito em virtude da metodologia envolvida na indução e no processamento do escarro, que é um método muito laborioso e que demanda tempo e pessoal altamente treinado para a sua realização.

A coloração para a contagem celular diferencial do escarro induzido, tanto na pesquisa como na prática clínica, tem sido feita utilizando a coloração May-Grünwald-Giemsa ou Wright-Giemsa. Essas colorações fazem parte de um grupo chamado de "colorações de Romanowsky".^(^ ^11^ ^)^ A coloração hematológica rápida (panótico rápido), também baseada na técnica de Romanowsky,^(^ ^12^ ^)^ utiliza reagentes similares, mas o tempo de coloração é consideravelmente menor; enquanto a coloração padrão necessita de 34 min, a coloração rápida é realizada em até 2 min. 

O encurtamento do tempo total de processamento seria muito importante para uma maior viabilidade da metodologia. Ainda, o custo da coloração hematológica rápida é consideravelmente menor quando comparada à coloração padrão. Por esses motivos, a validação dessa metodologia é importante, especialmente para países em desenvolvimento, como o Brasil.

O objetivo do presente estudo foi avaliar a confiabilidade da coloração hematológica rápida para a citologia de escarro induzido.

## Métodos

Foram incluídos no estudo 50 pacientes, entre os quais 21 pacientes asmáticos adultos que apresentaram asma não controlada, caracterizada por um escore do *Asthma Control Questionnaire**^(^* ^13^ ^)^ superior a 1,7 na última semana e confirmada objetivamente (nos últimos 3 anos) por limitação reversível ao fluxo de ar (aumento do VEF_1_ > 12% e VEF_1_ > 200 mL após a inalação de broncodilatador de curta ação) nos participantes com limitação ao fluxo de ar das vias aéreas (relação VEF_1_/CVF < 0,7); 19 portadores de DPOC com idade > 40 anos, história de sintomas respiratórios associados à obstrução moderada ou grave ao fluxo aéreo (VEF_1_ < 50% do previsto e relação VEF_1_/CVF < 0.7), em uso de qualquer tipo de medicação para DPOC e com história de tabagismo importante (atual ou prévia) com uma carga tabágica > 20 maços-ano; e 10 indivíduos assintomáticos para doenças respiratórias, caracterizados pela ausência de sintomas respiratórios, não fumantes e confirmados objetivamente por espirometria normal. Foram excluídos do estudo pacientes que tiveram infecção respiratória nas últimas quatro semanas, portadores de doenças graves de outros sistemas, portadores de outras doenças pulmonares conhecidas e mulheres grávidas.

O estudo foi realizado no Núcleo de Pesquisa em Asma e Inflamação das Vias Aéreas, localizado no Hospital Universitário da Universidade Federal de Santa Catarina, localizado na cidade de Florianópolis (SC), e devidamente aprovado pelo Comitê de Ética em Pesquisa com Seres Humanos da instituição (Processo no. 2093; FR 437236, emitido em 28/11/2011).Todos os participantes assinaram o termo de consentimento livre e esclarecido após uma explicação detalhada sobre o estudo.

Os participantes foram submetidos a espirometria antes e depois do uso de broncodilatador, de acordo com as especificações da *American Thoracic Society**^(^* ^14^ ^)^ com um espirômetro computadorizado (Koko; PDS Instrumentation, Inc., Louisville, CO, EUA). Os valores de referência utilizados foram os de Crapo et al.^(^ ^15^ ^)^

Posteriormente, foi realizada a indução do escarro de acordo com a metodologia descrita por Pizzichini et al.^(^ ^16^ ^)^ O procedimento envolveu a inalação de um aerossol de solução salina isotônica (0,9%) seguida pela inalação de aerossol de soluções salinas hipertônicas de forma seriada (3%, 4% e 5%) utilizando-se um nebulizador ultrassônico Fisoneb (Fisons, Pickering, Ontário, Canadá). A inalação do aerossol foi mantida por 1-2 min, de acordo com a gravidade da broncoconstrição presente antes do procedimento, seguida pela mensuração do VEF_1_. Os participantes foram orientados a enxaguar a boca, engolir a água e assoar o nariz para diminuir a contaminação por saliva ou descarga pós-nasal. A seguir, foram instruídos a tossir e a depositar o escarro em um recipiente limpo. Esses procedimentos foram consecutivamente repetidos, aumentando-se a concentração da solução a cada 7 min até o máximo de 21 min ou até que ocorresse uma queda do VEF_1_ ≥ 20%. 

O processamento do escarro foi iniciado em até 2 h após a coleta, que é o tempo máximo descrito na literatura.^(^ ^2^ ^)^ As porções densas do material expectorado foram selecionadas a olho nu ou sob visualização em microscópio invertido, separando o escarro da saliva. A fração selecionada foi tratada com DTT a 0,1% na proporção de quatro vezes do volume da fração. Essa mistura foi homogeneizada com uma pipeta de Pasteur e agitada por 15 min em agitador de mesa. Foi adicionado Dulbecco's PBS em quantidade correspondente a quatro vezes o volume inicial de escarro selecionado, e a suspensão resultante foi filtrada para a remoção de restos celulares e muco não dissolvido. Em seguida, foi realizada a contagem celular total de leucócitos em um hemocitômetro de Neubauer modificado, excluindo-se as células escamosas. A viabilidade celular foi determinada através do método de exclusão com *trypan blue*. A amostra foi ajustada para 1,0 × 106 células/mL, e foram confeccionadas quatro lâminas, que foram codificadas. Todas as lâminas do presente estudo foram confeccionadas por citocentrifugação. Depois de secarem ao ar livre, duas lâminas foram coradas por May-Grünwald-Giemsa, e as outras duas foram coradas pelo método em estudo (panótico rápido).

A coloração de May-Grünwald-Giemsa foi realizada pelo sistema automatizado Sysmex sp1000iTM (Sysmex Co., Kobe, Japão). Nesse método, as lâminas foram fixadas por imersão em metanol p.a., e em seguida, sua imersão em solução de corante de May-Grünwald e em solução de May-Grünwald diluída (1:1). Logo após, as lâminas foram imersas em solução de corante Giemsa fresca (diluição 1:10) e posteriormente secas. O procedimento completo durou exatamente 34 min, de acordo com a programação de rotina do equipamento recomendada pelo fabricante.

A coloração hematológica rápida foi feita manualmente. O processo iniciou-se com a imersão das lâminas na solução nº 1 (triarilmetano a 0,1%), mantendo-se um movimento contínuo para cima e para baixo por 5-10 s (5-10 imersões por 1 s cada). Em seguida, as extensões foram imersas na solução nº 2 (xanteno a 0,1%), repetindo o mesmo procedimento. Após escorrer, as lâminas foram imersas na solução nº 3 (tiazina a 0,1%), repetindo-se o mesmo procedimento. As lâminas foram enxaguadas com água destilada e deixadas ao ar livre para secagem.^(^ ^12^ ^)^ Essa técnica de coloração levou, no máximo, 2 min. Foram contadas 400 células não escamosas nas lâminas de cada coloração, de forma independente, por dois pesquisadores, qualificados para a leitura do escarro induzido. Como as lâminas estavam codificadas, isso impediu que os leitores das lâminas identificassem seus respectivos pares ou resultados de leituras anteriores. 

A confiabilidade do método foi aferida pelas concordâncias intraobservador e interobservador (coloração padrão vs. coloração teste do estudo) para as contagens celulares dos dois métodos por meio do coeficiente de correlação intraclasse (CCI), e foram utilizadas as representações gráficas de Bland & Altman.^(^ ^17^ ^)^ A interpretação do CCI foi baseada na classificação proposta por Landis e Koch.^(^ ^18^ ^)^ A concordância na identificação de escarro eosinofílico (eosinófilos ≥ 3%)(6) e de escarro neutrofílico (neutrófilos > 64%)^(^ ^19^ ^)^ entre observadores e entre as duas colorações foi calculada com estatística kappa.^(^ ^18^ ^)^ Diferenças entre as características dos grupos do estudo foram examinadas por ANOVA e pelo teste de Bonferroni na análise *post hoc*. Os testes estatísticos foram bicaudais, o nível de significância aceito foi de 5%, e para as análises, foi utilizado o pacote estatístico *Statistical Package for the Social Sciences*, versão 18.0 (SPSS Inc., Chicago, IL, EUA).

## Resultados

O processo de indução do escarro foi realizado em 62 indivíduos, dos quais 50 (80,6%) foram capazes de fornecer uma amostra adequada para leitura, com viabilidade celular superior a 50%. Doze amostras de escarro induzido foram consideradas inadequadas devido à elevada contaminação salivar (> 20% de células escamosas), à baixa viabilidade celular (< 50%) ou a insuficiência de material para a confecção das lâminas. As características demográficas, clínicas e funcionais dos participantes estão apresentadas na Tabela 1. Os grupos foram distintos e bem caracterizados, como demonstrado pelas diferenças demográficas, clínicas e funcionais.

**Tabela 1:** Características demográficas, clínicas e funcionais dos participantes.a

Características	Grupos			p
	Asma	DPOC	Controle	
	(n = 21)	(n = 19)	(n = 10)	
Idade, anos^b^	47,3 (22-68)	62,8 (52-77)	38,4 (21-58)	< 0,001*,*** e 0,2**
Gênero feminino^c^	12 (57,0)	5 (26,3)	7 (70,0)	0,03
VEF1 pré-BD,% previsto	55,3 ± 11,9	50,2 ± 18,2	102,2 ± 7,8	0,1* e < 0,001**,***
VEF1 pós-BD,% previsto	64,9 ± 11,7	52,7 ± 18,2	104,5 ± 8,7	0,02* e < 0,001**,***
VEF1/CVF pré-BD, %	58,3 ± 9,5	52,5± 13,8	80,4 ± 5,0	0,4* e < 0,001**,***
VEF1/CVF pós-BD,%	61,3 ± 9,2	53,2 ± 14,4	82,4 ± 4,6	0,06* e < 0,001**,***
ΔVEF1 pós-BD, L	0,29 ± 0,29	0,07 ± 0,09	0,08 ± 0,08	0,003*, 0,02** e 1,0***
ΔVEF1 pós-BD, %	19,1 ± 17,6	6,1 ± 7,4	2,5 ± 2,4	0,005*, 0,002** e 1,0***

Pré-BD : antes do uso de broncodilatador

pós-BD : depois do uso de broncodilatador

a Valores expressos em média ± dp, exceto onde indicado

b Valor expresso em média (mínimo-máximo)

c Valor expresso em n (%)

* Grupo asma vs. grupo DPOC

** Grupo asma vs. controle

*** Grupo DPOC vs. controle

As características celulares do escarro induzido foram as esperadas para os diferentes grupos do estudo. Quando comparados com portadores de DPOC e controles saudáveis, o escarro dos asmáticos caracterizou-se por uma proporção significativamente maior de eosinófilos. Em contraste, o escarro dos portadores de DPOC apresentou um aumento significativo na contagem celular total e na proporção de neutrófilos do que o do grupo controle. O grupo controle apresentou uma proporção significativamente maior de macrófagos que os outros dois grupos. A Figura 1 mostra as proporções de neutrófilos, eosinófilos e macrófagos nos diferentes grupos do estudo. Na Figura 2, as medianas das proporções de neutrófilos, eosinófilos e macrófagos, em toda a amostra estudada, estão separadas pelo tipo de coloração empregada. Observa-se que não houve diferenças entre as contagens das lâminas coradas pelos dois métodos utilizados.

**Figura 1:** Celularidade diferencial no escarro induzido nos três grupos do estudo.

**Figura 2:** Mediana da contagem celular diferencial em lâminas coradas com May-Grünwald-Giemsa (barra cinza escuro) e pela coloração hematológica rápida (barra cinza claro).

Os resultados da análise da concordância interobservador na contagem celular diferencial do escarro induzido nas lâminas coradas pelo método de May-Grünwald-Giemsa mostram que as medianas e percentis entre eles foram similares, e os CCI apontaram uma concordância quase perfeita para as leituras de eosinófilos (CCI = 1,00), neutrófilos (CCI = 0,99) e macrófagos (CCI = 0,98). A concordância para a leitura de linfócitos foi substancial (CCI = 0,76). Para as lâminas coradas pelo método da coloração hematológica rápida, as medianas e percentis entre observadores também ficaram muito próximas, e os CCI apontaram concordância quase perfeita para as contagens de eosinófilos (CCI = 1,00), neutrófilos (CCI = 0,99), macrófagos (CCI = 0,99) e linfócitos (CCI = 0,83).

Na análise de concordância intraobservador, os valores de CCI para os dois observadores nas contagens celulares diferenciais dos pares de lâminas coradas pelos dois métodos do estudo indicaram uma concordância quase perfeita para neutrófilos (CCI = 0,97 para ambos), eosinófilos (CCI = 0,99 e 0,98) e macrófagos (CCI = 0,96 para ambos). Nas contagens de linfócitos, a concordância do observador 1 foi substancial (CCI = 0,75), e a concordância do observador 2 foi moderada (CCI = 0,65). Estes resultados estão graficamente apresentados na Figura 3.^(^ ^17^ ^)^

**Figura 3:** Representações gráficas de Bland & Altman. Reprodutibilidade interobservador das proporções de neutrófilos (em A), eosinófilos (em B) e macrófagos (em C) no escarro induzido em lâminas coradas por May-Grünwald-Giemsa (MGG). Reprodutibilidade intraobservador das proporções de neutrófilos (em D), eosinófilos (em E) e macrófagos (em F) no escarro induzido em lâminas coradas pelos dois métodos de coloração. As representações se referem às diferenças entre as leituras dos observadores 1 e 2 (eixo y) em relação à média das leituras dos observadores 1 e 2 (eixo x). A linha interrompida central indica ausência de diferenças e as linhas interrompidas periféricas indicam dois desvios-padrão da média das diferenças. CCI: coeficiente de correlação intraclasse; NEU.: neutrófilos; EOS.: eosinófilos; e MAC.: macrófagos.

A concordância interobservador na identificação de escarro eosinofílico e neutrofílico nas lâminas coradas pelos dois métodos do estudo foi considerada de substancial a quase perfeita, conforme demonstrado na Tabela 2. No entanto, observa-se que a concordância intraobservador, embora considerada substancial, foi inferior àquela interobservador.

**Tabela 2:** Concordâncias interobservador e intraobservador para a identificação de escarro eosinofílico e escarro neutrofílico na avaliação de lâminas coradas pelos dois métodos do estudo.

Concordância interobservador	Tipo de escarro	kappa	p
Coloração hematológica rápida	Eosinofílico	1,000	< 0,001
	Neutrofílico	1,000	< 0,001
Coloração May-Grünwald-Giemsa	Eosinofílico	0,905	< 0,001
	Neutrofílico	0,960	< 0,001
Concordância intraobservador	Tipo de escarro	kappa	p
Observador 1	Eosinofílico	0,746	< 0,001
	Neutrofílico	0,801	< 0,001
Observador 2	Eosinofílico	0,758	< 0,001
	Neutrofílico	0,760	< 0,001

## Discussão

Os resultados do presente estudo mostram que as lâminas coradas pelo método de May-Grünwald-Giemsa e com o corante hematológico rápido permitem contagens celulares semelhantes, com altos índices de concordância intraobservador e interobservador. Esses resultados demonstram a confiabilidade do método da coloração hematológica rápida no processamento do escarro induzido. Esse fato é relevante porque o método da coloração hematológica rápida é mais simples, permite a redução no tempo de processamento do escarro em até 32 min sem prejudicar a qualidade da amostra, além de ser consideravelmente mais barato.

Até onde sabemos, o presente estudo foi o primeiro a analisar a confiabilidade do método da coloração hematológica rápida para a citologia de escarro induzido, comparando-o com um método de coloração tradicional, o método de May-Grünwald-Giemsa. A análise da confiabilidade e reprodutibilidade dos resultados é importante para confirmar a acurácia dos resultados obtidos usando a coloração hematológica rápida. No presente estudo, a análise da confiabilidade da coloração hematológica rápida foi testada por duas estratégias distintas. A primeira estratégia foi o cálculo do CCI para as contagens celulares por dois observadores independentes, impossibilitados de identificar as lâminas. Embora as colorações utilizadas no presente estudo pudessem ser identificadas pelo aspecto das lâminas, códigos diferenciados foram usados para impedir a identificação dos respectivos pares de lâminas. Estudos anteriores^(^ ^20^ ^-^ ^22^ ^)^ já haviam demonstrado que as concordâncias intraobservador e interobservador nas contagens de lâminas coradas com Wright-Giemsa e May-Grünwald-Giemsa podiam ser consideradas perfeitas, mas que isso dependia do grau de contaminação salivar das lâminas.^(^ ^21^ ^)^ Os resultados do presente estudo com a coloração hematológica rápida estão alinhados com os daqueles estudos. A segunda estratégia foi examinar a confiabilidade da coloração hematológica rápida na identificação de escarro eosinofílico e de escarro neutrofílico. Isso é relevante em função do emprego do exame de escarro na prática clínica para identificar fenótipos da asma grave, predizer a resposta ao tratamento e diminuir o número de exacerbações da asma por meio do controle da inflamação eosinofílica.

A análise da concordância entre os dois métodos de coloração na identificação de escarro eosinofílico ou neutrofílico, realizada por um mesmo observador (intraobservador) indicou que essa era substancial. Além disso, a concordância interobservador na identificação de escarro eosinofílico ou neutrofílico foi quase perfeita. Esses resultados novamente demonstram a confiabilidade da coloração hematológica rápida, por demonstrar sua acurácia na identificação dos diferentes fenótipos inflamatórios. Contudo, os resultados também mostraram que a concordância interobservador foi maior que a concordância intraobservador na identificação dos fenótipos. Essa diferença poderia ser explicada pela presença de variabilidade de conteúdo celular entre as lâminas coradas pelos dois métodos. Embora a concordância intraobservador tenha sido substancial, esse resultado em particular sugere cautela e necessidade de estudos posteriores para identificar a razão dessa variabilidade.

A reprodutibilidade das contagens diferenciais de leucócitos entre observadores em amostras de escarro induzido já havia sido relatada anteriormente.^(^ ^20^ ^-^ ^22^ ^)^ Em 1997, um grupo de autores obteve um alto índice de reprodutibilidade entre as leituras de observadores distintos para todos os tipos celulares estudados. Aqueles autores também encontraram um menor índice de concordância para os linfócitos, quando comparado com os dos demais tipos celulares. A menor concordância para as proporções de linfócitos foi considerada como consequência da presença de uma quantidade muito pequena desse tipo celular nas amostras de escarro induzido. No presente estudo, também encontramos menor concordância entre as contagens de linfócitos; porém, isso não foi enfatizado devido ao fato conhecido de que essas variações não têm relevância clínica.

Em um estudo,^(^ ^21^ ^)^ houve boa concordância interobservador para as contagens de neutrófilos, eosinófilos e macrófagos e, novamente, essa foi menor para as contagens de linfócitos; a justificativa para o menor índice de repetibilidade não foi relacionada apenas à escassez de linfócitos nas amostras mas também à dificuldade no reconhecimento dessas células. Um grupo de autores^(^ ^21^ ^)^ confirmou os resultados de um estudo anterior,^(^ ^20^ ^)^ demonstrando que a reprodutibilidade da análise diferencial da celularidade do escarro induzido é afetada pela contaminação salivar e pela baixa viabilidade celular. No presente estudo, amostras com contaminação salivar > 20% ou com viabilidade < 50% foram consideradas inadequadas para a análise.

Métodos de refinamento do exame do escarro em muito contribuíram para a sua acurácia e reprodutibilidade.^(^ ^23^ ^)^ Contudo, estudos anteriores focaram predominantemente nas etapas anteriores à obtenção das lâminas e à fase líquida do escarro.^(^ ^24^ ^-^ ^27^ ^)^

Em 2003, foi publicado um estudo que comparou os resultados e custos de três técnicas de análise do escarro induzido.^(^ ^28^ ^)^ As metodologias variaram entre a confecção de esfregaço a partir do escarro sem o tratamento com DTT, confecção de esfregaço do escarro tratado com DTT e confecção de lâminas do escarro tratado com DTT. Apesar de as primeiras duas técnicas reduzirem o tempo e o custo da análise do escarro, o coeficiente de correlação de Spearman pode ser considerado inadequado para esse tipo de análise, que variou de 0,57 a 0,64 para a contagem de eosinófilos e de 0,51 a 0,57 para a contagem de neutrófilos (p < 0,01 para ambos). Os autores concluíram que a técnica que utiliza lâminas de amostras tratadas com DTT e confeccionadas por citocentrifugação é mais indicada para fins de pesquisa e em centros especializados.^(^ ^28^ ^)^

Alguns dos aspectos que impedem o uso mais disseminado do escarro induzido são sua complexidade e o tempo de processamento das amostras. Dois estudos anteriores tentaram reduzir a complexidade do processamento das amostras,^(^ ^29^ ^,^ ^30^ ^)^ ambos usando um dispositivo de filtração de escarro (Accufilter; Cellometrics, Hamilton, Ontário, Canadá), que seria uma alternativa para a padronização do processamento do escarro induzido. O dispositivo consiste em um kit com tubo para a pesagem e tratamento do escarro selecionado da saliva, acoplado a um filtro e a um tubo receptor que contém DTT, solução salina e *trypan blue*. Um desses estudos^(^ ^30^ ^)^ procurou determinar a validade do uso desse dispositivo para a análise do escarro, comparando-o com a metodologia tradicional. Os resultados do estudo geraram CCIs que indicam uma boa reprodutibilidade entre as metodologias para contagens de eosinófilos e neutrófilos; entretanto, os CCI apontaram uma diminuição na viabilidade celular e na contagem total de células, assim como um aumento na proporção de células epiteliais escamosas com a utilização daquele dispositivo.

Em síntese, o elevado grau de concordância nas contagens celulares de lâminas coradas pelo método de May-Grünwald-Giemsa e com o corante hematológico rápido atesta a confiabilidade dessa última coloração, o que justifica sua recomendação para uso quando se pretende reduzir o tempo de processamento e os custos do exame do escarro induzido.
